# Utility of bronchoalveolar lavage in the management of immunocompromised patients presenting with lung infiltrates

**DOI:** 10.1186/s12890-019-0801-2

**Published:** 2019-02-26

**Authors:** Randall Choo, Naser Salman Hamza Naser, Nivedita Vikas Nadkarni, Devanand Anantham

**Affiliations:** 1 Duke-NUS Medical School, Singapore, Singapore; 2 Salmaniya Medical Complex, Manama, Bahrain; 3 Centre for Quantitative Medicine, Duke-NUS Medical School, Singapore, Singapore; 4 Department of Respiratory and Critical Care Medicine, Singapore General Hospital, Academia Building Level 3, 20 College Road, S169856, Singapore, Singapore

**Keywords:** Bronchoalveolar lavage, Flexible bronchoscopy, Immunocompromised, Lung infiltrates

## Abstract

**Background:**

Bronchoalveolar lavage (BAL) is utilized for diagnosing lung infiltrates in immunocompromised. There is heterogeneity in the data and reported diagnostic yields range from 26 to 69%. Therefore, selection criteria for BAL to maximize yield and minimize complications are unclear.

Objectives of this study were to determine the diagnostic yield and complication rate of BAL in immunocompromised patients presenting with lung infiltrates, and identify factors impacting these outcomes. Exploratory aims included characterization of pathogens, rate of treatment modification and mortality.

**Methods:**

Retrospective study from January 2012 to December 2016. Patients on mechanical ventilation were excluded. Positive diagnostic yield was defined as confirmed microbiological or cytological diagnosis.

**Results:**

A total of 217 patients were recruited (70.1% male and mean age: 51.7 ± 14.6 years). Diagnostic yield was 60.8% and complication rate 14.7%. Complications (hypoxemia and endobronchial bleeding) were all sell-limiting. Treatment modification based on BAL results was 63.3%. In 97.0% an infectious aetiology was identified. HIV infection (OR 5.304, 95% CI 1.611–17.458, *p* = 0.006) and severe neutropenia (OR 4.253, 95% CI 1.288–14.045, *p* = 0.018) were associated with positive yield. Leukemia (OR 0.317, 95% CI 0.102–0.982, *p* = 0.047) was associated with lower yield. No factors impacted complication rate. Overall mortality (90-day) was 17.5% and in those with hematologic malignancy, it was 28.3%.

**Conclusion:**

BAL retains utility in diagnosis of immunocompromised patients with lung infiltrates. However, patients with hematologic malignancy have a high mortality and alternative sampling should be considered because of poor results with BAL.

**Trial registration:**

ClinicalTrials.gov identifier NCT01374542. Registered June 16, 2011.

## Introduction

Lung infiltrates cause significant morbidity and mortality in immunocompromised patients [1, 2]. However, this is a heterogeneous group with various aetiology of underlying immunosuppression. The Infectious Diseases Society of America 2013 guidelines has included in its definition of highly immunocompromised patients the following groups: combined immunodeficiency disorder, chemotherapy for cancer, ≤ 2 months post-solid organ transplantation, human immunodeficiency virus (HIV) infection with a CD4 T-lymphocyte count < 200 cells/mm^3^, daily corticosteroid treatment with a dose ≥20 mg of prednisone or equivalent for ≥14 days and use of biologic immune modulators such as tumor necrosis factor-alpha blockers or rituximab [3]. Moreover in clinical practice, patients with hematological malignancies or neutropenia and those on steroid-sparing immunosuppressants are also considered immunocompromised.

Despite this heterogeneity of underlying etiology, the majority of these patients with lung infiltrates present in similar manner with cough, fever and dyspnea [4]. Empiric treatment is not without risks including adverse drug reactions, inadequacy of therapy and development of antimicrobial resistance. Therefore, obtaining a confirmed diagnosis is essential since the therapeutic paradigm varies widely depending on the cause of the lung infiltrates. In addition, early diagnosis has been associated with improved survival, with data reporting confirmed diagnosis within 5 days having a lower mortality compared to later diagnosis (32% vs. 51%, *p* = 0.024) [1].

Flexible bronchoscopy is commonly used for investigating lung infiltrates because it facilitates collection of microbiological and cytological samples via bronchoalveolar lavage (BAL) and can be performed in an ambulatory setting [5]. Complications of this procedure include hypoxemia and myocardial ischemia [5]. Data on diagnostic yield of BAL in immunocompromised patients presenting with lung infiltrates range from 26 to 69%; and complication rates range from 1 to 52% (Table 1). There is marked heterogeneity in the data both in inclusion criteria and study design. This makes it challenging to draw definite conclusions on which patients are most likely to benefit from BAL and on the prognosis of the various groups when categorized according to underlying cause of immunosuppression. In addition, there is limited data from countries where tuberculosis is endemic.

**Table 1:** Diagnostic yield and complication rate of BAL in immunocompromised patients presenting with lung infiltrates from a PubMed search since 2000

	Study design	Inclusion criteria	BAL Procedures (patient number)	Diagnostic yield %	Complication rate
Reichenberger et al., 2001 [16]	Retrospective	Post renal transplant	91 (71)	69% (63/91)	–
Hohenadel et al., 2001 [13]	Retrospective	Hematology patients	95 (95)	65% (62/95)	16% (15/95)
Rano et al., 2001 [17]	Prospective	Mixed etiology but HIV patients excluded	135 (200)	51% (68/135)	2% (3/135)
Taggart et al., 2002 [18]	Retrospective	HIV patients	216 (174)	50% (108/216)	–
Danés et al., 2002 [9]	Prospective	Mixed aetiology including HIV	134 (241)	52% (70/134)	–
Jain et al., 2004 [19]	Prospective	Mixed etiology, HIV excluded	99 (104)	38% (48/125)	14% (8/59)
Bissinger et al., 2005 [20]	Retrospective	Hematology patients	95 (77)	56% (53/95)	–
Peikert et al., 2005 [14]	Retrospective	Neutropenia	35 (35)	49% (17/35)	9% (3/35)
Hofmeister et al., 2006 [21]	Retrospective	Hematopoietic stem cell transplant	91 (78)	49% (45/91)	8% (7/91)
Vélez et al., 2007 [10]	Prospective	Mixed aetiology including HIV	109 (101)	49% (60/122)	–
Boersma et al., 2007 [22]	Prospective	Hematological malignancy	35 (32)	26% (9/35)	–
Burger, 2007 [23]	Retrospective	Hematopoietic stem cell transplant	27 (21)	52% (14/27)	52% (11/21)
Cordani et al., 2008 [24]	Prospective	Hematological malignancy	25 (24)	44% (11/25)	–
Hummel et al., 2008 [25]	Retrospective	Hematological malignancy	246 (199)	48% (118/246)	1% (3/249)
Shannon et al., 2010 [26]	Retrospective	Hematopoietic stem cell transplant	598 (501)	55% (329/598)	12% (74/598)
Sampsonas et al., 2011 [15]	Prospective	Mixed etiology but HIV patients excluded	284 (284)	34% (96/284)	4% (10/284)
Kottmann et al., 2011 [27]	Retrospective	Mixed etiology	190 (190)	56% (106/190)	–
Gilbert et al., 2012 [28]	Retrospective	Hematopoietic stem cell transplant	145 (144)	53% (77/145)	30% (49/162)
Brownback et al., 2013 [11]	Retrospective	Mixed aetiology including HIV	150 (133)	52.5% (79/150)	7% (11/150)
Kim et al., 2015 [29]	Retrospective	Hematologic malignancy	206 (187)	65% (134/ 206)	–
Svensson et al., 2017 [30]	Retrospective	Hematologic malignancy	151 (133)	39% (59/151)	13% (20/151)
Sakata et al., 2017 [31]	Retrospective	Hematopoietic stem cell transplant	179 (125)	40% (71/179)	–

The primary objective of this study was to determine the diagnostic yield of BAL in immunocompromised patients presenting with lung infiltrates. Patients who have already progressed to respiratory failure requiring mechanical ventilation have a mortality rate of nearly > 50% and need to be considered separately [1]. This population was beyond the scope of our study. Additionally, we aimed to determine the incidence of complications of BAL and identify factors associated with either higher diagnostic yields or lower complication rates. Exploratory aims include characterization of commonly isolated pathogens, the rate of post-procedure treatment modification as an indicator of clinical utility, as well as post-procedure 30 and 90-day mortality. Post-procedure treatment modification reflects the true value of the procedure since some microbiological findings may be of non-pathogenic commensals while other results may not be amendable to clinical intervention. Identification of such data will optimize patient selection, allowing endoscopists to counsel patients and recommend alternative diagnostic modalities for those with low likelihood of successful diagnosis or at high risk of complications.

## Materials and methods

This retrospective cross-sectional study evaluated immunocompromised patients with radiographic evidence of lung infiltrates who underwent BAL from January 2012 to December 2016 at a tertiary acute care hospital in Singapore. Inclusion criteria was (1) immunocompromised patients, (2) new (≤ 1 month) pulmonary infiltrates on chest radiograph or computed tomography scan, and (3) undergoing flexible bronchoscopy with BAL in an ambulatory setting. Immunocompromised patients were defined according to the Infectious Diseases Society of America 2013 guidelines as highly immunosuppressed or if they had either ongoing haematological malignancy, neutropenia or steroid-sparing immunosuppressant therapy [3]. Neutropenia severity was categorized based on absolute neutrophil count from mild (1000–1499 per microliter), moderate (500–999 per microliter) and severe (< 500 per microliter). Exclusion criteria comprised BAL performed in an ICU setting on mechanically ventilated patients, due to differences in procedure such as bronchoscope intubation via an endotracheal tube. Prognosis is also different and poor when the patient has progressed to respiratory failure [1]. Patients who underwent transbronchial lung biopsies or other forms of bronchoscopic sampling were also excluded to avoid introduction of confounding factors.

Data was extracted from a bronchoscopy database that prospectively collects, via endoscopists’ reports, all bronchoscopy details performed at the hospital endoscopy centre. This ensured integrity and completeness of data collection over the study period. Data was rendered non-identifiable with removal of patient’s name, identification card number and date of procedure. Chest computed tomography (CT) scans were interpreted by a radiologist and consultant pulmonologist and the predominant abnormalities characterized according to the following categories: consolidation, ground glass opacities, tree-in-bud appearance, reticular infiltrates, nodular infiltrates and cavitation. Institutional review board approval was obtained (SingHealth Centralised Institutional Review Board, reference number: 2011/350/C) and ClinicalTrials.gov identifier is NCT01374542. Waiver of consent was provided by SingHealth Centralised Institutional Review Board.

### Procedural details

Bronchoscopy was performed using an Olympus BF-1 T160 (Olympus, Tokyo, Japan) bronchoscope that has an outer diameter of 6.0 mm with a 2.8 mm working channel. All procedures were performed under moderate sedation using a combination of fentanyl and midazolam. BAL was obtained from the bronchopulmonary segment corresponding to CT scan findings for focal infiltrates while the right middle lobe or lingular was preferred in the cases with diffuse infiltrates. BAL samples were sent for standardized investigations consisting of cytology and microbiological analysis.

Microscopy for bacteria was performed with Gram stain, acid fast bacilli with Ziehl-Neelsen stain and *Pneumocystis jirovecii* with Gomori methenamine silver. This was followed by bacterial, fungal and mycobacterial cultures. Polymerase chain reaction testing was performed for *Mycobacterium tuberculosis* (TB-Protec) and respiratory viruses: influenza, parainfluenza, respiratory syncytial virus, coronavirus, adenovirus, rhinovirus and metapneumovirus. Cytomegalovirus detected by BAL fluid antigen assay or cell-based virus isolation was considered pathogenic only in the presence of either intracellular inclusion bodies on cytology or concomitant positive serum antigenemia. Galactomannan antigen was performed via enzyme-linked immunosorbent assay.

### Outcome measures

Primary outcome was diagnostic yield as defined as the number of BAL with a positive diagnostic study divided by the total number of patients. A positive study was defined as either a confirmed diagnosis on cytology or microbiology. Post-BAL treatment modification was considered positive if treatment was documented to be initiated, escalated or discontinued in response to diagnostic BAL findings and if treatment change was in keeping with BAL findings. Patients were followed-up for a minimum of 6 months. Sub-group analysis was performed according to underlying cause of immunosuppression and patients were classified into 3 groups: HIV positive patients, ongoing hematological malignancy and others (HIV negative, non-hematologic malignancy).

Complications were considered associated with bronchoscopy if they occurred peri-procedurally or within 24 h after the patient underwent the procedure. Complication rate was calculated as the number of procedures with complications divided by the total number of procedures performed. Complications were classified into six categories using Common Terminology Criteria for Adverse Events (CTCAE) definitions/severity of pneumothorax, airway bleeding, hypoxia, hypotension and requirement for escalation of level of care [6].

### Statistical analysis

Statistical analyses were performed using statistical software SPSS (IBM Corp. Released 2016. IBM SPSS Statistics for Windows, Version 24.0. Armonk, NY: IBM Corp). Findings were considered statistically significant for all analyses with *p*-value < 0.05. Continuous variables were summarized using mean (standard deviation, SD) or median (interquartile range, IQR) and categorical variables were summarized using frequency (%). Fisher’s exact test and Student’s t test or Mann-Whitney U test as appropriate were used to compare categorical and continuous variables respectively. Secondary outcomes were analyzed using logistic regression. Factors with unadjusted odds ratios from univariate logistic regression that satisfied the criteria of *p*-value < 0.2 were included in multivariable logistic regression analysis to determine the adjusted odds ratios of factors significantly associated with diagnostic yield and complication rate.

## Results

Flexible bronchoscopy with BAL was performed on 217 immunocompromised patients between January 2012 to December 2016. One hundred fifty-two patents (70.1%) were male and the mean age was 51.7 ± 14.6 years. Fifty-nine patients (27.2%) were HIV positive amongst whom 3 had concurrent lymphoma with 2 receiving ongoing chemotherapy. All 59 were analyzed under the HIV category because that was the underlying cause of immunosuppression. A further 92 patients (92/217; 42.4%) had an ongoing hematologic malignancy: 55 with leukemia, 28 with lymphoma, 6 with myelodysplastic syndrome and 3 with multiple myeloma. Among patients with hematologic malignancy, 75 (75/92; 81.5%) had received chemotherapy within the preceding 6 months, 60 patients (60/92; 65.2%) were neutropenic at the time of presentation of pulmonary infiltrates and 19 (19/92; 20.7%) had received hematopoietic stem cell transplants. The remaining 66 (66/217; 30.4%) HIV negative, non-hematologic malignancy patients had immunosuppression due to other causes: of whom 27 received chemotherapy for solid organ malignancies, 15 had solid organ transplants, 38 had received steroid-sparing immunosuppressants and 14 had received high dose corticosteroid therapy.

Fever was the most common presenting symptom (67.3%, 146/217) followed by cough (53.5%, 116/217) and dyspnea (36.4%, 79/217). Median duration of symptoms was 14 days (IQR 8–22.5). Most patients (88.5%, 192/217) received empiric antibiotics prior to bronchoscopy for a median duration of 5 days (IQR 2–10). CT chest scan was obtained in 83.4% (181/217) of patients, with consolidation being the most common finding (66.9%, 121/181) followed by ground glass opacities (51.3%, 93/181). CT scans were performed with a median of 3 days (IQR 2, 5) prior to BAL procedure. Patient characteristics are presented in Table 2. Median duration of bronchoscopy was 10 min (IQR 10–15) and the following sedation was used: median midazolam 2.50 mg (IQR 2.00–3.50) and fentanyl 50 microgram (IQR 25–50). The median volume of BAL fluid instilled was 120 mL (IQR 100–140) and > 30% of instilled volume was retrieved in 91% of cases (152/167).

**Table 2:** Patient characteristics

	n (%)
Total number of patients	217
Mean age in years	51.7 ± 14.6
Male gender	152 (70.0%)
Current Smokers	27 (12.4%)
Cause of immunosuppression:	
HIV/ AIDS	59 (27.2%)
High dose corticosteroid	16 (7.4%)
Steroid-sparing immunosuppressive medication	44 (20.3%)
Solid organ transplant	15 (6.9%)
Bone marrow transplant	33 (15.7%)
Hematologic malignancy	95 (43.8%)
Leukemia	55 (25.3%)
Lymphoma	28 (12.9%)
Myelodysplastic syndrome	6 (2.8%)
Multiple myeloma	3 (1.4%)
Chemotherapy	104 (47.9%)
Neutropenia	72 (33.2%)
Co-morbidities:	
Cardiac (e.g. heart failure, ischemic heart disease)	9 (4.1%)
Pulmonary (e.g. asthma, chronic obstructive pulmonary disease)	23 (10.6%)
Hepatic (e.g. chronic hepatitis, cirrhosis)	21 (9.7%)
Renal (e.g. chronic kidney disease)	13 (6.0%)
Treatment at the time of BAL:	
Antibiotics	192 (88.5%)
Median duration, days (IQR)	5 (2 - 10)
Antivirals	103 (47.5%)
Antifungals	83 (38.2%)
Clinical presentation:	
Fever	146 (67.3%)
Cough	116 (53.5%)
Dyspnea	79 (36.4%)
Pleurisy	19 (8.8%)
Median symptom duration, days (IQR)	14 (8–22.5)
Radiologic presentation on CT:	*n* = 181
Consolidation	121 (66.9%)
Ground glass opacities	93 (51.3%)
Nodules	63 (34.8%)
Cavitation	20 (11.0%)
Tree-in-bud appearance	18 (9.9%)
Reticular pattern	11 (6.1%)

Overall diagnostic yield for this study was 60.8% (132/217). Post-procedure treatment modification based on BAL results was 63.3% (84/132). Majority of positive BAL yielded infectious agents (97.0%, 128/132) with 37 cases of *Pneumocystis jirovecii,* 13 *Mycobacteria tuberculosis* and 12 rhinovirus. Cytomegalovirus was found in 16 cases but no inclusion bodies were identified on cytology. Table 3 shows all BAL microbiological results. Galactomannan testing was also positive in 31 cases, with ≥0.5 antigen index considered positive. Non-infectious causes of pulmonary infiltrates was found in 4 cases based on cytology and clinical/radiological presentation: eosinophilic pneumonia, squamous cell carcinoma, lung adenocarcinoma and drug-induced pneumonitis. Differences in characteristics of patients with positive and negative BAL are outlined in Table 4.

**Table 3:** BAL microbiology results

	n (%)
Bacterial	
Total	43
*Mycobacterium tuberculosis*	13 (30.2%)
*Pseudomonas aeruginosa*	8 (18.6%)
*Klebsiella* spp.	5 (11.6%)
*Enterococcus* spp.	4 (9.3%)
*Stentotrophomonas maltophilia*	4 (9.3%)
*Staphylococcus aureus*	2 (4.7%)
*Acinetobacter baumannii*	1 (2.3%)
*Escherichia coli*	1 (2.3%)
*Mycobacterium abscessus*	1 (2.3%)
*Mycobacterium avium*	1 (2.3%)
*Nocardia* sp.	1 (2.3%)
*Streptococcus pneumoniae*	1 (2.3%)
*Streptococcus anginosus*	1 (2.3%)
Fungal Microscopy/Culture	
Total	51
*Pneumocystis jirovecii*	37 (72.5%)
*Aspergillus* spp.	10 (19.6%)
*Cryptococcus* spp.	3 (5.9%)
*Phaeoacremonium* spp.	1 (2.0%)
Viral	
Total	64
Cytomegalovirus	16 (25.0%)
Rhinovirus	12 (18.8%)
Coronavirus	9 (14.1%)
Parainfluenza	9 (14.1%)
Influenza	7 (10.9%)
Respiratory syncytial virus	7 (10.9%)
Adenovirus	3 (4.7%)
Metapneumovirus	1 (1.6%)

**Table 4:** Factors impacting BAL diagnostic yield (^*^p < 0.05)

	Negative BAL n (%)	Positive BAL n (%)	p-value
Total	85 (39.2%)	132 (60.8%)	
Male gender	54 (63.5%)	98 (74.2)	0.098
Mean age in years ± SD	52.4 ± 14.6	51.3 ± 14.6	0.588
Current smokers	10 (11.8%)	17 (12.9%)	1.000
Cause of immunosuppression:			
HIV/ AIDS	12 (14.1%)	47 (35.6%)	0.001^*^
High dose corticosteroids	8 (9.4%)	8 (6.1%)	0.428
Steroid-sparing immunosuppressive medication	17 (20.0%)	27 (20.5%)	1.000
Hematologic malignancy	46 (54.1%)	49 (37.1%)	0.017^*^
Leukemia	30 (35.3%)	25 (18.9%)	0.010^*^
Lymphoma	14 (16.5%)	17 (12.9%)	0.552
Myelodysplastic	2 (2.4%)	4 (3.0%)	1.000
Multiple myeloma	0 (0%)	3 (2.3%)	0.282
Chemotherapy	54 (63.5%)	50 (37.9%)	< 0.001^*^
Neutropenia	32 (37.6%)	40 (21.6%)	0.302
Mild (ANC 1000–1499/μl)	8 (9.6%)	4 (3.1%)	0.127
Moderate (ANC 500–999/μl)	7 (8.4%)	6 (4.6%)	
Severe (ANC < 500/μl)	16 (19.3%)	29 (22.1%)	
Clinical presentation:			
Fever	60 (74.1%)	86 (65.6%)	0.224
Cough	38 (46.9%)	78 (59.5%)	0.089
Dyspnea	26 (32.1%)	53 (40.5%)	0.244
Pleurisy	7 (8.6%)	12 (9.2%)	1.000
Median symptom duration in days (IQR)	15 (8–66)	19 (11–65.5)	0.017^*^
Treatment at the time of BAL:			
Antibiotics	75 (90.4%)	117 (90.0%)	1.000
Median duration antibiotics in days (IQR)	7 (4, 10)	6 (3, 11)	0.477
Antifungals	33 (38.8%)	50 (37.9%)	0.887
Antivirals	43 (50.6%)	60 (45.5%)	0.488
Radiologic presentation on CT (*n* = 181):			
Consolidation	44 (54.3%)	57 (57.0%)	0.764
Ground glass opacities	42 (51.9%)	51 (51.0%)	1.000
Tree-in-bud appearance	11 (13.6%)	7 (7.0%)	0.211
Reticular pattern	3 (3.7%)	8 (8.0%)	0.350
Nodules	32 (39.5%)	31 (31.0%)	0.273
Cavitation	6 (7.5%)	14 (14.0%)	0.233
BAL segments:			
Upper lobes	44 (51.8%)	62 (47.7%)	0.579
Right middle lobe	26 (30.6%)	32 (24.6%)	0.350
Lower lobes	32 (37.6%)	53 (40.8%)	0.671

In multivariable logistic regression analysis, factors significantly associated with higher diagnostic yield included HIV infection (adjusted OR 5.304, 95% CI 1.611–17.458, *p* = 0.006), severe neutropenia (adjusted OR 4.253, 95% CI 1.288–14.045, *p* = 0.018) and presence of cavitatory lesions on chest CT scan (adjusted OR 3.824, 95% CI 0.877–16.680, *p* = 0.074). In addition, leukaemia as an underlying cause of immunosuppression was significantly associated with lower diagnostic yield (adjusted OR 0.317, 95% CI 0.102–0.982, *p* = 0.047) (See supplementary materials in Appendix 1). In univariate analysis, haematological malignancy (unadjusted OR 0.501, 95% CI 0.288–0.871, *p* = 0.014) and ongoing chemotherapy at the time of the scope (unadjusted OR 0.350, 95% CI 0.199–0.616, *p* < 0.001) had a lower diagnostic yield (Appendix 1).

Overall complication rate for this study was 14.7% (32/217) with 94% (30/32) attributable to self-limiting hypoxemia that required supplemental oxygen therapy temporarily (CTCAE 2). The remaining two cases were of endobronchial bleeding that did not require further endoscopic intervention (CTCAE 1). Both patients were thrombocytopenic with platelet counts of 27,000 per microliter and 126,000 per microliter. No patients required intubation or escalation of care. Post-procedure 30-day and 90-day mortality was 10.6% (23/217) and 17.5% (38/217) respectively. Patients who suffered complications had significantly higher median Charlson comorbidity index (median 5, IQR 3–7) compared to those who did not suffer complications (median 4, IQR 3–6; *p* = 0.011) [7]. Univariate logistic regression analysis for complication rate found dyspnea during disease presentation to be significantly associated with increased complications (unadjusted OR 2.508, 95% CI 1.169–5.382, *p* = 0.018). Multivariable logistic regression analysis found no factors significantly associated with complication rate (See supplementary material in Appendix 2).

### Subgroup analyses

Diagnostic yield of BAL in HIV patients was 79.7% (47/59) of which the majority of positive diagnoses was of *Pneumocystis jirovecii* (70.2%, 33/47). Other diagnoses included 4 cases of *Mycobacteria tuberculosis*, 4 rhinovirus and 3 *Aspergillus* species. Despite the high diagnostic yield, only 40.4% (19/47) of BAL results in HIV directly impacted patient management. Complication rate was 20.3% (12/59) all of which was attributable to hypoxemia requiring supplemental oxygen. Post-procedure 30-day and 90-day mortality was 1.7% (1/59) and 6.8% (4/59) respectively.

In non-HIV, hematologic malignancy patients diagnostic yield was 51.1% (47/92) and of these 70.2% (33/47) of cases impacted patient management with treatment modification. Isolated pathogens included 8 cases of parainfluenza virus, 5 *Pseudomonas aeruginosa*, 4 rhinovirus, 4 respiratory syncytial virus, 3 *Mycobacterium tuberculosis*, 3 *Klebsiella* species and 3 coronavirus. Complication rate was 10.9% (10/92), while post-procedure 30-day and 90-day mortality was 17.4% (16/92) and 28.3% (26/92) respectively. Mortality rate was significantly higher for non-HIV, hematologic malignancy patients than for HIV and for non-HIV, non-hematologic malignancy groups.

In non-HIV, non-hematologic malignancy patients diagnostic yield was 57.6% (38/66), with 84.2% (32/38) of these resulting in modification of therapy. Pathogens included 6 cases of *Mycobacterium tuberculosis*, 5 human coronavirus, 5 *Aspergillus* species, 4 *Pneumocystis jirovecii*, 4 rhinovirus and 3 cases of *Pseudomonas aeruginosa*. Complication rate was 15.2% (10/66) and post-procedure 30-day and 90-day mortality was 9.1% (6/66) and 12.1% (8/66) respectively. Test of proportions indicated that mortality rate was significantly different in at least one category (HIV patients, non-HIV hematologic malignancy patients or non-HIV, non-hematologic malignancy patients) compared to the others (*p* = 0.008).

## Discussion

The diagnostic yield of BAL in immunocompromised patients presenting with lung infiltrates in an ambulatory setting was 60.8% and in the majority of cases, the positive BAL findings impacted clinical management. Infectious etiologies accounted for 97% of the positive diagnoses which reinforces current clinical practice of early use of empiric antibiotics. In addition, our study found no impact of prior anti-microbial therapy or duration of antibiotics on diagnostic yield. The mismatch between positive yield and post-BAL result treatment modification may be attributable to the fact that 40.5% (64/158) of pathogens detected were respiratory viruses for which therapeutic options were limited. In addition, in none of the 16 isolates of cytomegalovirus was there inclusion bodies detected in the corresponding cytology. This raises doubts over the pathogenic nature of the cytomegalovirus isolates. Cases where the BAL cytomegalovirus isolate was the only finding (including no antigenemia) were not labelled as positive diagnostic yield. There were 17.1% (37/217) cases of *Pneumocystis jirovecii*, 6.0% (13/217) cases of tuberculosis, 4.6% (10/217) cases of Aspergillosis and 3.7% (8/217) cases of *Pseudomonas* infection. In addition, in 14.3% (31/217) BAL Galactomannan was positive.

We also showed differences in the diagnostic yield between different groups based on causes of underlying immunosuppression. HIV patients had the highest diagnostic yield and the majority of positive *Pneumocystis jirovecii* results identified were in this group. It also meant that post-BAL treatment modification was the lowest in this group because Pneumocystis pneumonia was often the clinical diagnosis and appropriate empiric treatment had already been commenced. There was also a trend towards increased risk of post procedure hypoxia in HIV patients which endoscopists should note with respect to ensuring appropriate post-procedure monitoring. Previous data on HIV patients showed a diagnostic yield of 50–60% [8], and mixed groups with HIV patients showed yields of 49 to 52.5% [9–11].

In contrast, the diagnostic yield was lower in patients with hematologic malignancy, especially those with leukaemia. There are possible explanations for this. Non-infective aetiology such as alveolar haemorrhage may be more prevalent in this group. BAL can only procure cytological and not histological specimens for analysis. Other conditions such as drug induced pneumonitis may not have pathognomonic findings and it may be difficult to establish a confirmed cause when it is a diagnosis of exclusion. Therefore, alternative sampling including lung biopsies should be considered early in patients with hematologic malignancies.

Non-HIV, non-hematologic malignancy patients had a diagnostic yield that was in between the HIV patient and hematologic malignancy group. The most pertinent finding in this group was that the most commonly identified pathogen was *Mycobacteria tuberculosis*. This may be reflective of the endemic nature of tuberculosis in our population in Singapore [12], as well as the nature of the immunosuppression. This finding is corroborated by the presence of radiological evidence of cavitation in 11% of our patients and the trend towards increased diagnostic yield if cavitation was indeed present.

Complication rate was significant at 14.7% but was entirely self-limiting. There was a trend towards a higher rate in patients with higher co-morbidity burden and in those who were hypoxic at presentation. Co-morbidities are likely to impact the effect of moderate sedation on the cardio-respiratory system. Hypoxemia may be exacerbated by the introduction of saline for lavage and the degree of decruitment from bronchoscopic suctioning.

Our study also confirmed earlier data that prognosis of immunocompromised patient with pulmonary infiltrates was guarded and varied with underlying cause of immunosuppression. The all-cause 30-day and 90-day mortality ranged from 1.7 and 6.8% in HIV patients to 17.4 and 28.3% respectively in those with hematologic malignancy. Prior data showed a 30-day mortality in haematology patients to be 22% [13] and in neutropenic patients to be 26% [14]. In a mixed aetiology study, the 30-day mortality was 7% in non-hematologic malignancy and 19% with hematologic malignancy [15]. This reflects the severity of illness that immunocompromised patients with pulmonary infiltrates have and should serve as an impetus to continue to improve bronchoscopic sampling and laboratory testing. Symptoms and radiological findings (besides the trend in presence of cavitation) also did not impact diagnostic yield in our study. Therefore, identifying patients who are unlikely to get a bronchoscopic diagnosis on the basis of only clinical presentation may be challenging. Delaying diagnosis due to a negative BAL result may also risk clinical deterioration.

Limitations of this study include the retrospective design. However, the fact that the data was extracted from a prospective bronchoscopy database directly populated by endoscopists’ reports ensured completeness of data. In addition, procedure details were collected in a standardized format. Sample size may also have lacked statistical power to detect factors associated with complications because of the low complication rate. Finally, this data was limited to a single institution study. However, this meant that BAL sampling and microscopic testing was performed in a standardized manner.

## Conclusion

Flexible bronchoscopy with BAL retains a role in the management of immunocompromised patients presenting with lung infiltrates especially in establishing a confirmed microbiological diagnosis. A diagnosis is possible in 60.8% and the complications are largely self-limiting. However, the diagnostic yield varies with underlying cause of immunosuppression and alternative sampling should be considered early in patients with hematologic malignancies because of poor results with BAL. This group with haematologic malignancy has a high 90-day mortality and delayed diagnosis risk clinical deterioration. Tuberculosis appears to be an important pathogen in endemic regions especially in the non-HIV, non-hematologic malignancy group. Aspergillus was the other commonly identified pathogen via culture and Galactomannan assay. Viruses accounted for 40.5% of positive diagnosis and this finding often leads to limited modification in clinical management. Our data show both the utility and limitations of BAL in the approach to immunocompromised patients presenting with lung infiltrates. This data will serve as the foundation on which newer therapeutic strategies such as transbronchial cryobiopsy can be evaluated.

## Appendix 1

**Table 5:** Univariate and multiple logistic regression for diagnostic yield (^*^*p* < 0.05)

	Univariate OR (95% CI)	*p*-value	Multiple OR (95% CI)	*p*-value
Male Gender	1.655 (0.918–2.983)	0.094		
Mean age in years	0.995 (0.976–1.014)	0.586		
Current smokers	1.109 (0.482–2.552)	0.808		
Cause of immunosuppression:				
High dose corticosteroids	0.621 (0.224–1.723)	0.360		
HIV/ AIDS	3.364 (1.659–6.820)	0.001^*^	5.304 (1.611–17.458)	0.006^*^
Steroid-sparing immunosuppressive medication	1.029 (0.522–2.029)	0.935		
Solid organ transplant	0.963 (0.330–2.811)	0.946		
Bone marrow transplant	1.217 (0.567–2.610)	0.614		
Hematologic malignancy	0.501 (0.288–0.871)	0.014^*^		
Leukemia	0.428 (0.230–0.798)	0.008^*^	0.317 (0.102–0.982)	0.047^*^
Lymphoma	0.750 (0.348–1.614)	0.461		
Myelodysplastic	1.297 (0.232–7.240)	0.767		
Multiple myeloma	N.A.	N.A.		
Chemotherapy	0.350 (0.199–0.616)	< 0.001^*^		
Neutropenia	0.720 (0.405–1.279)	0.263		
Severity (Reference no neutropenia)				
Mild (ANC 1000–1500/μl)	0.283 (0.081–0.984)	0.047	0.249 (0.42–1.460)	0.123
Moderate (ANC 500–999/μl)	0.214 (0.155–1.518)	0.214	1.749 (0.385–7.942)	0.469
Severe (ANC < 500/μl	1.024 (0.509–2.060)	0.946	4.253 (1.288–14.045)	0.018^*^
Clinical Presentation:				
Fever	0.669 (0.362–1.236)	0.199		
Cough	1.665 (0.952–2.912)	0.074		
Dyspnea	1.437 (0.803–2.574)	0.222		
Pleurisy	1.066 (0.402–2.830)	0.898		
Symptom duration	1.021 (1.001–1.042)	0.039		
Treatment at the time of BAL:				
Antibiotics	0.960 (0.380–2.426)	0.931		
Duration of antibiotics	0.995 (0.964–1.028)	0.784		
Antifungals	0.961 (0.549–1.683)	0.889		
Antivirals	0.814 (0.471–1.405)	0.460		
Radiologic Presentation on CT:				
Consolidation	1.115 (0.618–2.010)	0.718		
Ground glass opacities	0.966 (0.538–1.737)	0.909		
Tree-in-bud appearance	0.479 (0.177–1.298)	0.148		
Reticular pattern	2.261 (0.580–8.815)	0.240		
Nodules	0.688 (0.372–1.272)	0.233		
Cavitation	2.031 (0.743–5.554)	0.167	3.824 (0.877–16.680)	0.074
Segments:				
Upper lobes	0.850 (0.492–1.468)	0.559		
Right middle lobe	0.741 (0.403–1.364)	0.335		
Lower lobes	1.140 (0.650–1.998)	0.647		
Number of pulmonary segments sampled: ≥ 2 (reference one segment)	0.516 (0.270–0.987)	0.045^*^	0.347 (0.125–0.965)	0.043^*^

## Appendix 2

**Table 6:** Univariate and multiple logistic regression for risk of BAL complications (^*^*p* < 0.05)

	Univariate OR (95% CI)	*p*-value	Multiple OR (95% CI)	*p*-value
Male gender	2.029 (0.793–5.194)	0.140		
Mean age in years	0.986 (0.961–1.011)	0.263		
Current smokers	1.372 (0.479–3.933)	0.556		
Cause of immunosuppression:				
High dose corticosteroids	0.366 (0.47–2.869)	0.338		
HIV/ AIDS	1.762 (0.801–3.876)	0.159		
Steroid-sparing immunosuppressive medication	1.120 (0.450–2.789)	0.808		
Solid organ transplant	1.491 (0.396–5.610)	0.554		
Bone marrow transplant	0.738 (0.241–2.258)	0.595		
Hematologic malignancy	0.535 (0.240–1.192)	0.126		
Leukemia	0.500 (0.182–1.370)	0.178		
Lymphoma	0.580 (0.165–2.034)	0.395		
Myelodysplastic	N.A.	N.A.		
Multiple myeloma	12.267 (1.079–139.514)	0.043^*^		
Chemotherapy	0.821 (0.386–1.748)	0.609		
Neutropenia	0.902 (0.402–2.022)	0.802		
Severity (reference no neutropenia)				
Mild (ANC 1000–1500/ml)	1.848 (0.464–7.372)	0.384		
Moderate (ANC 500–999/ml)	0.462 (0.057–3.736)	0.469		
Severe (ANC < 500/ml)	0.853 (0.323–2.255)	0.749		
Comorbidities				
Diabetes mellitus	0.893 (0.343–2.324)	0.816		
Cardiac	0.714 (0086–5.908)	0.754		
Pulmonary	1.248 (0.395–3.942)	0.706		
Hepatic	1.412 (0.442–4.505)	0.560		
Renal	1.810 (0.470–6.974)	0.388		
Charlson comorbidity index	1.108 (0.948–1.295)	0.196		
Clinical Presentation:				
Fever	1.184 (0.515–2.722)	0.690		
Cough	1.708 (0.778–3.749)	0.182		
Dyspnea	2.508 (1.169–5.382)	0.018^*^		
Pleurisy	0.639 (0.140–2.911)	0.563		
Duration of symptoms	1.005 (0.988–1.022)	0.592		
Treatment at the time of BAL:				
Antibiotics	N.A.	N.A.		
Duration of Antibiotics	0.960 (0.903–1.021)	0.195		
Antifungals	0.489 (0.208–1.146)	0.100		
Antivirals	0.839 (0.394–1.786)	0.649		
Radiologic presentation on CT:				
Consolidation	1.417 (0.610–3.292)	0.418		
Ground glass opacity	2.107 (0.891–4.980)	0.090		
Tree-in-bud appearance	N.A.	N.A.		
Reticular pattern	2.281 (0.565–9.208)	0.247		
Nodules	0.376 (0.135–1.048)	0.061	0.391 (0.140–1.093)	0.074
Cavitation	0.596 (0.130–2.728)	0.505		
Duration of procedure in minutes	1.009 (0.954–1.068)	0.742		
Dose of midazolam in milligram	1.001 (0.999–1.004)	0.386		
Dose of fentanyl in microgram	1.002 (0.987–1.018)	0.792		
Segments:				
Upper lobes	0.892 (0.420–1.893)	0.766		
Right middle lobe	0.725 (0.295–1.779)	0.482		
Lower lobes	1.227 (0.574–2.621)	0.597		
Number of pulmonary segments sampled: ≥ 2 (reference one segment)	0.701 (0.301–1.633)	0.410		

## Abbreviations

BAL: Bronchoalveolar lavage
CI: Confidence interval
CT: Computed tomography
CTCAE: Common Terminology Criteria for Adverse Events
HIV: Human immunodeficiency virus
ICU: Intensive care unit
IQR: Interquartile range
OR: Odds ratio
SD: Standard deviation
